# Genome-Wide Identification and Characterization of the Maize *ZmGT14* Gene Family Reveals ZmGT14-36 as a Drought-Responsive Gene Interacting with UGT85A2

**DOI:** 10.3390/plants15030512

**Published:** 2026-02-06

**Authors:** Minghao Sun, Yunlong Li, Sinan Li, Erna Wu, Yue Yin, Yan Sun, Shujun Li, Yuyang Duan, Xin Li, Quan Cai, Jianguo Zhang

**Affiliations:** 1 Maize Research Institute, Heilongjiang Academy of Agricultural Sciences, Harbin 150086, China; sunminghao_yg@yeah.net (M.S.); 13945699869@163.com (Y.L.); mrlee890323@163.com (S.L.); wu_erna@163.com (E.W.); yin-yue-yy@163.com (Y.Y.); sunyan19850301@163.com (Y.S.); lshj_750425@163.com (S.L.); andrea8929@sina.com (Y.D.); maize_lee@163.com (X.L.); 2 Postdoctoral Innovation Practice Base, Maize Research Institute, Heilongjiang Academy of Agricultural Sciences, Harbin 150028, China; 3 Key Laboratory of Biology and Genetics Improvement of Maize in Northern Northeast Region, Ministry of Agriculture and Rural Affairs, Harbin 150086, China

**Keywords:** *ZmGT14*, maize, *UGT85A2*, drought stress

## Abstract

Drought stress significantly disrupts plant water balance and cell wall integrity, thereby inhibiting growth and development. The Glycosyltransferase 14 (GT14) family plays a pivotal role in cell wall biosynthesis and environmental stress responses; however, the mechanisms underlying its involvement in the drought response of maize (*Zea mays* L.) remain elusive. In this study, we identified 42 *ZmGT14* members distributed across 10 chromosomes by genome-wide analysis. Phylogenetic relationships, gene structures, and conserved motif analyses indicated high intra-subfamily conservation. Promoter analysis revealed that *ZmGT14* genes are enriched with various stress-responsive elements, including ABRE, DRE, and MBS. Transcriptomic profiling and RT-qPCR verification demonstrated that the expression of *ZmGT14-36* increased by approximately 30-fold within 36 h of drought treatment. Further screening and point-to-point Yeast Two-Hybrid (Y2H) assays identified that ZmGT14-36 physically interacts with UGT85A2, a protein associated with redox homeostasis. These findings provide preliminary evidence that *ZmGT14-36* may participate in the drought resistance response in maize. Collectively, our study elucidates the molecular evolutionary characteristics of the *ZmGT14* family and provides a key candidate gene for the molecular breeding of drought-tolerant maize varieties.

## 1. Introduction

Due to their sessile nature, plants are inevitably exposed to a wide range of abiotic and biotic stresses, including drought, salinity, and low-temperature stress, throughout their growth and development [1]. Among these, drought stress represents one of the most severe environmental constraints on crop productivity [2], as it causes extensive cellular dehydration and loss of turgor pressure, ultimately compromising cellular structural integrity [3,4]. To survive under fluctuating environmental conditions, plants have evolved intricate physiological and molecular defense mechanisms [5,6]. The plant cell wall not only serves as a physical barrier against external stresses but also functions as a primary interface for environmental signal perception. Dynamic remodeling of cell wall structure and composition plays a pivotal role in plant stress responses [7].

Plant cell walls consist of a dynamic and intricate architectural network formed by the interweaving of cellulose, hemicelluloses, and pectins [8,9]. Embedded within this matrix are arabinogalactan proteins (AGPs), a diverse and ubiquitous class of hydroxyproline-rich glycoproteins. By interacting with pectic and hemicellulosic polysaccharides, AGPs contribute significantly to cell wall reinforcement and serve as pivotal mediators in both biotic and abiotic stress signaling [10]. The structural complexity of AGPs is characterized by a tripartite organization: an N-terminal signal peptide, a central glycomodular domain enriched in Pro-Ala-Ser-Thr (PAST) motifs, and a C-terminal glycosylphosphatidylinositol (GPI) anchor signal. The GPI anchor is essential for the initial targeting of AGPs to the endoplasmic reticulum and their subsequent translocation to the plasma membrane [11]. Recent advancements have elucidated that AGP glycan chains are synthesized via extensive post-translational modifications within the secretory pathway, orchestrated by the coordinated action of multiple glycosyltransferases (GTs). Specifically, members of the glycosyltransferase family 14 (GT14) function as glucuronosyltransferases, catalyzing the transfer of glucuronic acid (GlcA) residues onto the β-1,3- and/or β-1,6-linked galactan backbones of AGPs This glycosylation step is critical for the maturation of the structurally elaborate glycan chains that define AGP function [12,13].

Upon the perception of environmental stimuli, a rapid influx of extracellular calcium ions (Ca^2+^) is elicited, triggering a sophisticated cascade of physiological and biochemical responses [14,15,16]. Accumulating evidence suggests that AGPs, owing to their unique glycan architectures, are capable of stoichiometric binding and release of extracellular Ca^2+^, thereby functioning as dynamic calcium reservoirs or “calcium capacitors” [17,18]. Under stress-induced activation, the Ca^2+^ mobilized from AGPs serves as a critical second messenger to prime downstream defense systems [18]. Recent advancements further indicate that *GT14* family members may assemble into multienzyme complexes with other glycosyltransferases, such as UDP-glycosyltransferases (UGTs), to coordinately orchestrate the biosynthesis of these complex AGP glycan chains. The disruption of *GT14* function can directly impair the sequestration and mobilization of the extracellular calcium pool, ultimately manifesting as developmental abnormalities or compromised stress resilience [19].

Despite the essential role of *GT14* in AGP biosynthesis, comprehensive investigations of this gene family remain relatively limited. Current research has gradually shifted from the characterization of individual enzymatic activities toward elucidating the complex regulatory networks underlying plant stress adaptation [20]. In *Arabidopsis thaliana*, five *GT14* members (*AtGlcAT14A*-*E*) have been functionally characterized as glucuronosyltransferases (GlcATs) that incorporate glucuronic acid (GlcA) residues into the galactan side chains of AGPs. Notably, *glcat14a/b/e* triple mutants exhibit a severe reduction in GlcA content and fail to survive beyond the seedling stage, highlighting the indispensable role of *GT14* mediated glycosylation in fundamental plant growth and development [12,21,22].

Phylogenetic analyses across Arabidopsis (*Arabidopsis thaliana*), rice (*Oryza sativa*), and poplar (*Populus trichocarpa*) have revealed that the Domain of Unknown Function 266 (*DUF266*) shares high sequence similarity with the conserved *GT14* catalytic core, leading to its classification within the *GT14*-like subfamily [23,24]. In rice, the DUF266-containing protein BC10 is crucial for maintaining cell wall integrity; loss of *BC10* function markedly reduces arabinose levels in cellulose and hemicellulose fractions, thereby weakening mechanical strength and resulting in a pronounced dwarf phenotype [20]. Moreover, the Golgi-localized OsGT14;1 plays a pivotal role in plant growth and productivity. The *Osgt14;1* mutant displays pleiotropic defects, including shortened roots, reduced plant stature, brittle culms, and decreased grain yield, which are accompanied by substantial reductions in cellulose content and altered compositions of matrix polysaccharides, such as xylose and arabinose [25].

Maize (*Zea mays* L.) stands as a quintessential global staple crop, serving as a primary source of food, animal feed, and industrial feedstock. Consequently, maintaining yield stability in maize is a strategic pillar for ensuring global food security [26,27]. However, the escalating frequency and intensity of drought episodes, exacerbated by shifting climatic patterns, have emerged as formidable environmental constraints on maize growth, development, and yield potential [28,29]. In this study, we performed a comprehensive genome-wide identification and characterized 42 members of the *ZmGT14* gene family in maize. We systematically investigated their physicochemical properties, gene architectures, phylogenetic relationships, and cis-regulatory landscapes. By integrating transcriptomic profiling with RT-qPCR validation, we prioritized *ZmGT14-36* as a core candidate gene involved in drought-stress orchestration. Furthermore, the physical interactome of *ZmGT14-36* was explored through yeast library screening and point-to-point yeast two-hybrid (Y2H) assays. Collectively, our findings provide a novel theoretical framework and essential genetic resources for elucidating the molecular mechanisms by which cell wall-associated processes facilitate drought resilience, thereby advancing molecular breeding strategies for climate-resilient maize.

## 2. Materials and Methods

### 2.1. Plant Materials and Drought Treatment

Maize inbred line B73 was maintained and propagated in our laboratory. Seeds were soaked in distilled water for 24 h and then sown in plastic pots filled with a vermiculite: soil mixture (1:1, *v*/*v*). Plants were grown in a controlled growth chamber under the following conditions: 25 °C/22 °C (day/night) with a 16 h/8 h light/dark photoperiod. When seedlings reached the three-leaf stage (approximately one month after sowing), drought stress was imposed using 25 mL of 20% (*w*/*v*) polyethylene glycol 6000 (PEG 6000, Coolaber, Beijing, China) per tube [30]. Leaf samples were collected at 0, 1, 3, 6, 12, 24, 36, and 48 h after PEG treatment. Approximately 0.1 g of leaf tissue was harvested for each sample. Three independent biological replicates were collected at each time point, and each biological replicate was analyzed with three technical replicates. All samples were immediately frozen in liquid nitrogen and stored at −80 °C until further analysis.

### 2.2. Identification and Physicochemical Property Analysis of the ZmGT14 Gene Family

The maize reference genome assembly (Zm-B73-REFERENCE-NAM-5.0), along with corresponding protein sequences and annotation files, were downloaded from the Phytozome database (https://phytozome-next.jgi.doe.gov/, accessed on 1 July 2025). To identify *GT14* family members in maize, a hidden Markov model (HMM) search was performed against the maize protein database using HMMER v3.3, with the *GT14* conserved domain profile (PF02485) obtained from the Pfam database [31]. In parallel, known GT14 protein sequences from Arabidopsis and rice were used as queries to conduct BLASTP searches against the maize protein dataset. An E-value threshold of 1 × 10^−10^ was used for both BLASTP (version 2.15.0) and HMM (version 3.4) searches. Candidate gene IDs obtained from both approaches were combined and subjected to further validation. The presence of conserved *GT14* domains was subsequently confirmed using SMART (http://smart.embl.de/, accessed on 1 July 2025), the NCBI Conserved Domain Database (CDD; https://www.ncbi.nlm.nih.gov/cdd/, accessed on 1 July 2025), and Interpro (https://www.ebi.ac.uk/interpro/, accessed on 1 July 2025). Sequences lacking the core GT14 domain were excluded, and the remaining sequences were designated as bona fide *ZmGT14* family members.

The physicochemical properties of the identified ZmGT14 proteins, including molecular weight and theoretical isoelectric point, were analyzed using the ProtParam online tool (https://web.expasy.org/protparam/, accessed on 1 July 2025). Subcellular localization of ZmGT14 proteins was predicted using DeepLoc-2.0 (https://services.healthtech.dtu.dk/services/DeepLoc-2.0/, accessed on 1 July 2025).

### 2.3. Chromosomal Localization and Synteny Analysis

Based on genome annotation information, the physical locations of *ZmGT14* genes on the 10 maize chromosomes were visualized using TBtools v2.376 [32]. Phylogenetic analysis of the *GT14* family from maize, rice, and *Arabidopsis thaliana* was performed using MEGAX (version 10.2) software. A neighbor-joining (NJ) tree was constructed based on multiple sequence alignments generated with ClustalW (version 2.1), using the Poisson substitution model. The reliability of the phylogenetic tree was evaluated by bootstrap analysis with 1000 replicates [33]. The resulting phylogenetic tree was further visualized and annotated using Evolview v3 (https://www.evolgenius.info/evolview/, accessed on 15 July 2025). Intraspecific synteny analysis of the maize genome was conducted using TBtools. In addition, genome data for rice and *Arabidopsis thaliana* were retrieved to perform interspecies synteny analyses between maize and these two species.

### 2.4. Conserved Domain and Gene Structure Analysis of ZmGT14 Gene Family

Conserved motifs in ZmGT14 proteins were identified using the MEME Suite v5.5.9 (https://meme-suite.org/meme/index.html, accessed on 15 July 2025) with the following parameters: minimum motif width of 6, maximum motif width of 100, and the number of motifs set to 15. Gene structure information for *ZmGT14* family members, including exon–intron organization, was extracted from the maize genome annotation file and visualized using TBtools.

### 2.5. Cis-Acting Element Analysis of ZmGT14 Gene Promoters

The 2 kb upstream sequences of all *ZmGT14* genes were extracted from the maize genome based on the genome sequence and annotation files to represent their promoter regions. These sequences were submitted to the PlantCARE database for identification of *cis*-acting regulatory elements [34]. The results were subsequently visualized using R (version 4.3.1), following the approach described by Zhang [35].

### 2.6. Expression Analysis of ZmGT14 Genes Under Abiotic Stress

Transcriptome data related to abiotic stress were retrieved from the NCBI SRA database (PRJNA952945). Gene expression quantification was performed using Salmon V1.10.3 [36], and transcripts per million (TPM) values for *ZmGT14* family members were extracted. Heatmaps of gene expression profiles were generated using TBtools.

### 2.7. Quantitative Real-Time PCR (RT-qPCR) and Data Analysis

Total RNA was extracted from leaf tissues using the TransZol UP reagent kit(TransGen, Beijing, China) and reverse-transcribed into cDNA following the manufacturer’s instructions. Gene-specific primers were designed using NCBI Primer-BLAST (https://www.ncbi.nlm.nih.gov/tools/primer-blast/, accessed 6 August 2025) (Table S1), and *ZmActin* was used as an internal reference gene (Table S1) [37]. Quantitative real-time PCR was performed on a QuantStudio 3 instrument using TransScript TOP Green qPCR SuperMix(TransGen, Beijing, China). The thermal cycling conditions were as follows: initial denaturation at 94 °C for 30 s, followed by 40 cycles of denaturation at 94 °C for 5 s and annealing/extension at 60 °C for 30 s. Relative gene expression levels were calculated using the 2^−ΔΔCt^ method [38]. Statistical analyses were conducted with SPSS (version 22), and data visualization was performed using GraphPad Prism (version 10.1.2).

### 2.8. Gene Ontology (GO) Annotation and Enrichment Analysis

Functional annotation of the maize proteome at the genome-wide level was performed using the EggNOG-mapper (http://eggnog5.embl.de/#/app/home (accessed on 10 August 2025)) online tool to obtain Gene Ontology (GO) information for *ZmGT14* family members. GO enrichment analysis of the identified *ZmGT14* genes was subsequently conducted using the R package ClusterProfiler (v4.0), covering the three GO categories: Biological Process (BP), Cellular Component (CC), and Molecular Function (MF) [39]. Enriched terms related to stress responses were visualized using TBtools or the ggplot2 (version 3.5.1) package in R.

### 2.9. Yeast Two-Hybrid (Y2H) Screening

Bait construction and autoactivation test: The full-length CDS of *ZmGT14-36* was cloned into the pGBKT7 vector (Table S1). The resulting bait plasmid was co-transformed with the empty pGADT7 vector into Y2H yeast competent cells using the PEG/LiAc method. Transformants were plated on SD/-Leu/-Trp (DDO), SD/-Leu/-Trp/-His (TDO), and SD/-Leu/-Trp/-His/-Ade (QDO) media to assess colony growth, thereby excluding autoactivation activity or cytotoxicity of the bait protein. Library screening and validation: The pGBKT7-*ZmGT14-36* bait strain was co-transformed with a maize nuclear cDNA library plasmid and plated on TDO medium for initial screening. The maize nuclear cDNA library used in this study was previously constructed and maintained in our laboratory. Colony-forming units (CFUs) were counted to evaluate library coverage. Positive clones from the initial screen were re-streaked onto QDO medium supplemented with X-α-Gal to select stable interacting clones that grew normally and exhibited blue coloration. Clone identification and sequencing: Candidate positive single clones were subjected to colony PCR amplification using T7/AD-R primers. PCR products with single, clear bands were purified and sequenced. Obtained sequences were compared against the NCBI database using BLAST (https://blast.ncbi.nlm.nih.gov/Blast.cgi, accessed on 10 September 2025) to identify potential interacting proteins of *ZmGT14-36*.

### 2.10. Yeast Two-Hybrid Validation

To verify the physical interactions between *ZmGT14-36* and candidate proteins, the full-length CDSs of *ZmGT14-36*, *Zm00001eb020690*, *Zm00001eb087380*, and *Zm00001eb330890* were cloned into the pGBKT7 and pGADT7 vectors (Table S1), respectively. The resulting constructs were co-transformed into yeast two-hybrid (Y2H) competent cells. Transformants were plated on SD/-Leu/-Trp (DDO) and SD/-Leu/-Trp/-His/-Ade (QDO) selective media. Growth on QDO medium was used to determine whether direct protein–protein interactions occurred between ZmGT14-36 and the candidate proteins.

## 3. Results

### 3.1. Identification and Synteny Analysis of ZmGT14 Genes

**Figure 1:**
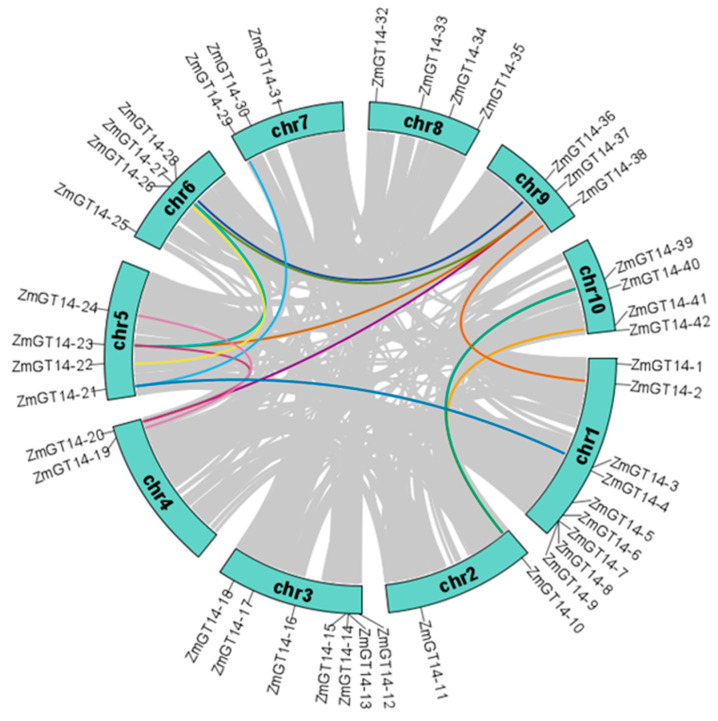
Chromosomal distribution and synteny analysis of *ZmGT14* genes in maize. The colored blocks (chr1–chr10) represent the ten maize chromosomes. The specific physical locations of the 42 *ZmGT14* members are indicated on the periphery of the chromosomes. The interior grey lines illustrate syntenic blocks across the entire maize genome, while the highlighted colored lines specifically denote segmental duplication events between *ZmGT14* gene pairs.

Using a combination of HMMER (PF02485) and BLASTP searches, we screened the maize (*Zea mays* B73) genome for *GT14* family members. Candidate protein sequences obtained from both methods were further validated for the presence of *GT14* conserved domains using CDD, SMART, and Interpro. This process led to the identification of 42 bona fide *ZmGT14* genes. Based on their physical positions on the chromosomes, these genes were systematically named *ZmGT14-1* to *ZmGT14-42* (Table S2). The chromosomal distribution and syntenic relationships of *ZmGT14* genes were visualized using TBtools (Figure 1), revealing 13 intra-genomic syntenic gene pairs.

Synteny analysis indicated that *ZmGT14* family members exhibit distinct one-to-many and many-to-one duplication patterns. For instance, several *ZmGT14-23* genes on chromosome 5 and *ZmGT14-37* on chromosome 9 were involved in multiple inter-chromosomal syntenic pairings, representing one-to-many duplication events. Conversely, a typical many-to-one pattern was observed for *ZmGT14-40* and *ZmGT14-42* on chromosome 10, both of which are syntenic with *ZmGT14-10* on chromosome 2 (Table S3). These results suggest that following whole-genome duplication events, *ZmGT14* family members underwent differential gene retention and loss during genome rearrangement.

### 3.2. Physicochemical Properties of ZmGT14 Gene Family

Physicochemical properties of the *ZmGT14* gene family were analyzed, revealing notable variation in protein length, molecular weight (MW), charge properties, and predicted subcellular localization among members (Table S2). Protein lengths ranged from 144 to 464 amino acids (corresponding CDS lengths of 432–1392 bp), indicating substantial structural diversity within the family. Molecular weights ranged from 17.07 to 51.00 kDa, with the majority of proteins concentrated in the 40–50 kDa range. The predicted isoelectric points (pI) varied widely from 5.26 to 11.60, reflecting significant differences in acidic and basic properties; for example, ZmGT14-5 and ZmGT14-31 are strongly basic, whereas ZmGT14-9 and ZmGT14-40 are acidic. All ZmGT14 proteins exhibited negative GRAVY values (−0.64 to −0.01), suggesting an overall hydrophilic nature consistent with the biochemical characteristics of glycosyltransferases functioning in the endomembrane system.

Predicted subcellular localization further indicated potential functional differentiation among family members. Most proteins were predicted to localize to the Golgi apparatus, aligning with their roles in glycosylation of cell wall polysaccharides such as AGPs (Table S2). Some members were predicted to localize to the endoplasmic reticulum (ZmGT14-1, ZmGT14-18, ZmGT14-28), cytoplasm (ZmGT14-27, ZmGT14-30), or extracellular space (ZmGT14-2, ZmGT14-5). Notably, ZmGT14-9 was predicted to localize in the nucleus, suggesting potential functional divergence from other family members.

### 3.3. Motif Composition and Gene Structure of ZmGT14 Gene Family

**Figure 2:**
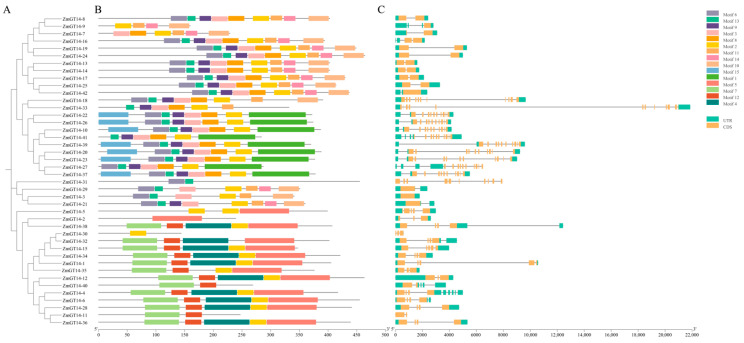
Phylogenetic relationships, conserved motif composition, and gene structure of *ZmGT14* family members. The figure consists of three components: (**A**) shows a phylogenetic tree constructed based on full-length protein sequences; (**B**) displays motif distribution, with 15 conserved motifs (Motif 1−15) indicated by colored blocks; (**C**) shows gene structures, where green blocks represent untranslated regions (UTR), yellow blocks represent coding sequences (CDS), and grey lines indicate introns.

To further investigate the structural features of the *ZmGT14* gene family and their relationship with evolutionary divergence, motif composition and gene structure analyses were conducted. Based on protein sequence similarity and phylogenetic relationships, the 42 *ZmGT14* members were classified into four major subfamilies (I–III) (Figure 2A). Members within the same subfamily exhibited high evolutionary conservation.

Motif analysis revealed distinct motif distribution patterns among subfamilies (Figure 2B). For example, subfamily I contained motifs 6, 13, 9, 3, 8, 2, 5, and 11; subfamily II contained motifs 6, 13, 9, 3, 8, and 2; subfamily III contained motifs 15, 1, 10, 3, 8, 2, and 5; subfamily IV generally contained a conserved combination of motifs 1, 4, 7, 11, and 12.

Gene structure analysis (Figure 2C, Table S4) showed that exon numbers ranged from 1 (*ZmGT14-3*) to 14 (*ZmGT14-27*), while intron numbers varied from 0 to 13. Most subfamily members exhibited highly consistent structural patterns; for instance, members of group I generally contained 10–13 introns. Notably, *ZmGT14-3* is an intronless gene with a single exon, suggesting a potential intron-loss event during evolution. This high diversity in gene structure likely underlies the functional diversification of the *ZmGT14* gene family during its evolutionary history.

### 3.4. Cis-Acting Elements in the Promoters of ZmGT14 Genes

**Figure 3:**
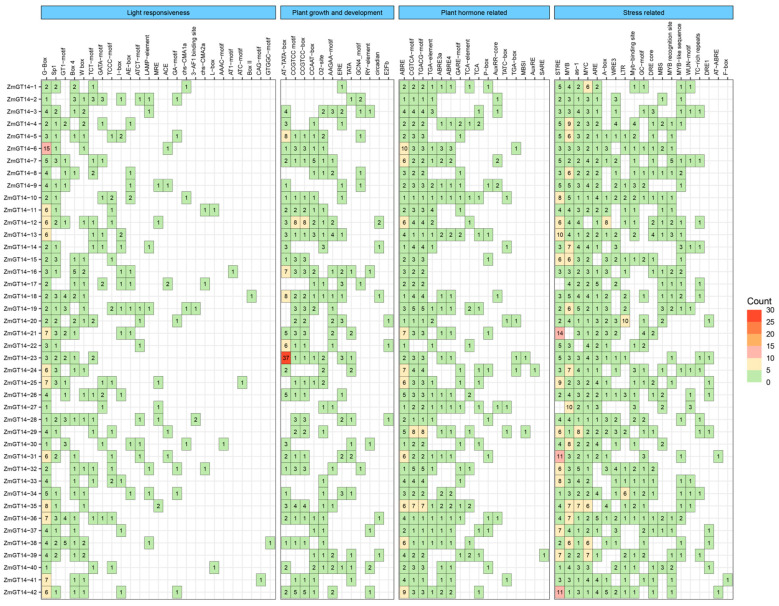
Analysis of *cis*-acting elements in the promoters of *ZmGT14* genes. The four blue headers at the top categorize the cis-elements based on their predicted functions: light responsiveness, plant growth and development, hormone responsiveness, and stress responsiveness. The heatmap uses a color gradient to indicate the number of each element, with green representing fewer copies, red representing more copies, and blank cells indicating the absence of the element in the promoter of a given gene.

Prediction and analysis of promoter sequences for the 42 *ZmGT14* genes revealed that *cis*-acting elements could be grouped into four major categories (Figure 3): light responsiveness, growth and development, hormone responsiveness, and stress responsiveness. Although core elements such as G-box, ABRE, MYB, AS-1, MYC, and ARE were commonly present across the family, significant variation in element distribution was observed among individual genes. In growth- and development-related elements, *ZmGT14-23* was enriched for AT~TATA-box elements (37 copies). Within the light-responsive category, *ZmGT14-6* contained the highest number of G-box elements (15 copies), and multiple G-box motifs were also identified in *ZmGT14-11*, *12*, *13*, *21*, *24*, *25*, *31*, *35*, *36*, *41*, and *42*. For hormone-responsive elements, ABRE motifs were widely distributed in all members, with higher copy numbers in *ZmGT14-6*, *7*, *12*, *21*, *24*, *25*, *32*, *35*, and *42*. Additionally, the presence of MBSI (*ZmGT14-23*, *29*), AuxRE (*ZmGT14-25*), and SARE (*ZmGT14-39*) suggests that *ZmGT14* genes may integrate multiple hormonal signals to coordinate responses to stress. Promoters of *ZmGT14* genes were also enriched in stress-responsive elements, including DRE, LTR, and STRE (drought, low temperature, and general stress), WUN-MOTIF and AS-1 (wounding and pathogen response), as well as MYB/MYC motifs. These findings further support a critical role for the *ZmGT14* family in mediating plant responses to environmental stresses.

### 3.5. Phylogenetic Analysis of ZmGT14 Gene Family

**Figure 4:**
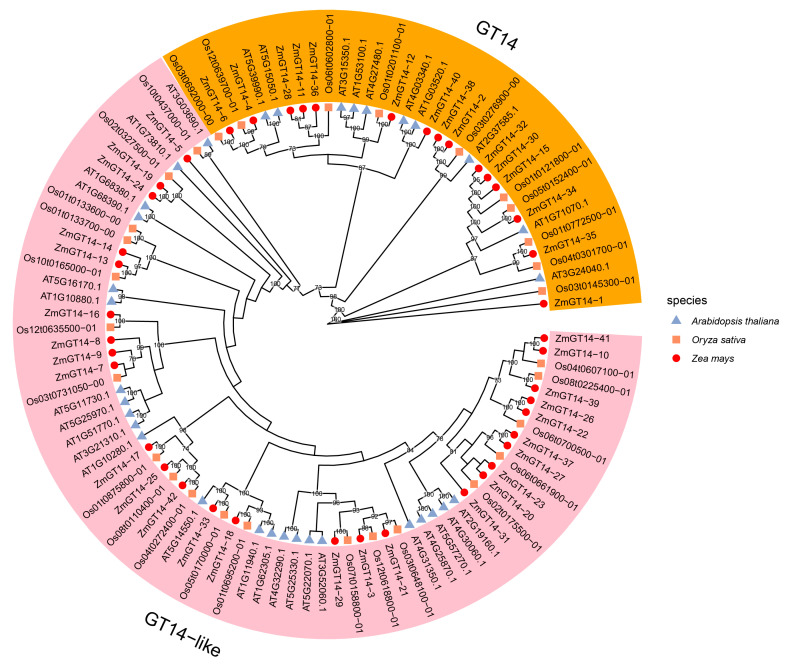
Phylogenetic analysis of GT14 proteins from maize, rice, and Arabidopsis. The phylogenetic tree illustrates the evolutionary relationships of GT14 proteins from maize, rice, and Arabidopsis. Three geometric symbols distinguish the species: red circles represent maize, orange squares represent rice, and blue triangles represent Arabidopsis. Based on sequence similarity, the entire family was divided into two major clades: *GT14* (orange background) and *GT14*-like (pink background). Bootstrap values are indicated at the nodes.

To clarify the evolutionary relationships and potential functional divergence of *ZmGT14* members, a phylogenetic tree was constructed using protein sequences from maize, *Arabidopsis thaliana*, and rice (Figure 4). Following the classification criteria established for the *DUF266* domain-containing gene family [23], the 42 *ZmGT14* members were clearly divided into two subgroups: *GT14* and *GT14*-like. Phylogenetic analysis revealed an uneven distribution of *ZmGT14* members across the two clades, with 15 members in the *GT14* clade and 27 members in the *GT14*-like clade. Many *ZmGT14* genes clustered with homologous rice genes, reflecting the high evolutionary conservation of this family in monocotyledonous plants.

### 3.6. Synteny Analysis of GT14 Gene Family

**Figure 5:**
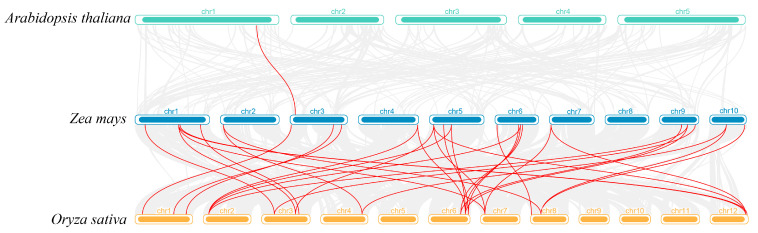
Synteny analysis of *ZmGT14* genes among maize, Arabidopsis, and rice. The figure illustrates the genomic synteny relationships of *ZmGT14* genes between maize and Arabidopsis as well as rice. Gray lines represent syntenic blocks across the whole genome, while red lines specifically highlight syntenic pairs between *ZmGT14* family members and their homologous genes.

To elucidate the evolutionary origins and interspecific homology of the *ZmGT14* gene family, synteny comparison maps were constructed between maize and both Arabidopsis and rice (Figure 5). Only a single syntenic gene pair (*ZmGT14-13/AT1G68380.1*) was identified between maize and Arabidopsis. In contrast, maize and rice exhibited a high degree of syntenic conservation, with 25 orthologous gene pairs identified, broadly distributed across seven maize chromosomes. Further analysis revealed that certain rice genes, such as *Os12t0618800-01*, corresponded to multiple copies in maize (chr1, 5, 7), whereas some maize genes, including *ZmGT14-3*, were associated with multiple rice homologs. These complex one-to-many or many-to-many relationships suggest that the *ZmGT14* family has undergone substantial segmental duplications or whole-genome duplication events during evolution, providing a basis for subsequent functional diversification.

### 3.7. miRNA-Mediated Regulation of ZmGT14 Genes

**Figure 6:**
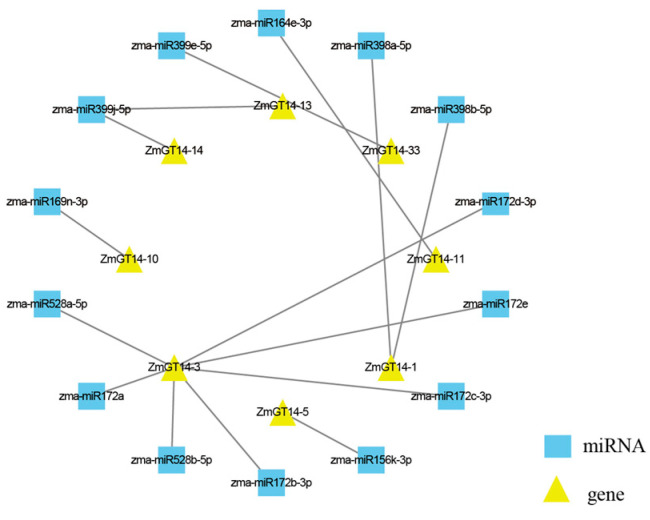
Predicted miRNA regulatory network of *ZmGT14* genes. Blue squares represent predicted miRNA nodes, while yellow triangles indicate their target *ZmGT14* gene nodes. Gray lines between nodes denote predicted interactions.

Beyond transcriptional regulation, miRNA-mediated mechanisms are crucial for plants to precisely respond to environmental signals. To investigate post-transcriptional control, a miRNA-target regulatory network for the *ZmGT14* gene family was predicted and constructed (Figure 6). The analysis revealed that 14 zma-miRNA family members target 8 *ZmGT14* genes, indicating a complex regulatory pattern. Among them, the zma-miR172 family (comprising five members: a, b, c, d, and e) occupies a central regulatory role, predominantly targeting *ZmGT14-1* and *ZmGT14-3*, which are also coordinately regulated by zma-miR528. Additionally, zma-miR398 specifically targets *ZmGT14-33*, while zma-miR164 and zma-miR399 regulate *ZmGT14-13* and *ZmGT14-14*, respectively. These highly conserved miRNAs, including miR172, miR528, and miR398, are known to play broad roles in plant growth, development, and stress responses [40,41,42]. The establishment of this regulatory network indicates that *ZmGT14* genes are not only controlled at the transcriptional level via diverse cis-elements but are also fine-tuned post-transcriptionally through miRNA-mediated cleavage or translational repression, enabling precise responses to environmental stimuli.

### 3.8. Predicted Protein–Protein Interaction Network of ZmGT14 Gene Family

**Figure 7:**
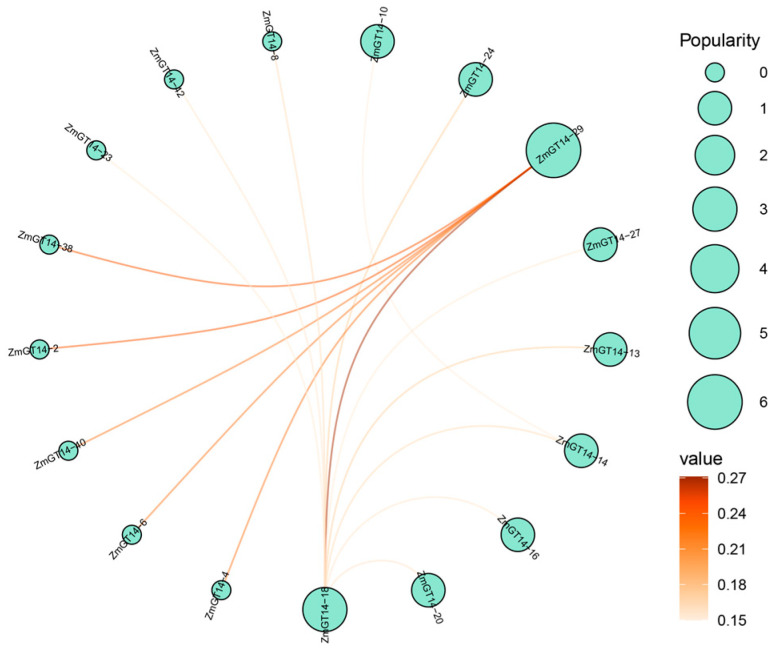
Predicted protein–protein interaction (PPI) network of *ZmGT14* gene family. Nodes (circles) represent individual ZmGT14 proteins, with node size (popularity) reflecting connectivity or centrality within the network. Edges between nodes indicate predicted interactions, and the edge color intensity (value) represents the confidence level of the interactions.

During cellular processes, glycosyltransferases often form multi-enzyme complexes to enhance catalytic efficiency or achieve substrate specificity [43,44]. To explore potential interactions among ZmGT14 family members, a protein–protein interaction (PPI) network was predicted (Figure 7). In the network, ZmGT14-29 exhibited the highest connectivity (popularity) and interaction strength (value), showing significant strong interactions (dark orange edges) with multiple members including ZmGT14-2, 4, 6, 38, and 40. In addition, ZmGT14-18 acted as another central node, mediating interactions with ZmGT14-14, 16, 20, and others. This highly centralized interaction pattern suggests that ZmGT14 proteins tend to form homo- or hetero-oligomeric complexes, likely facilitating their biological functions.

### 3.9. Gene Ontology (GO) Annotation of ZmGT14 Gene Family

**Figure 8:**
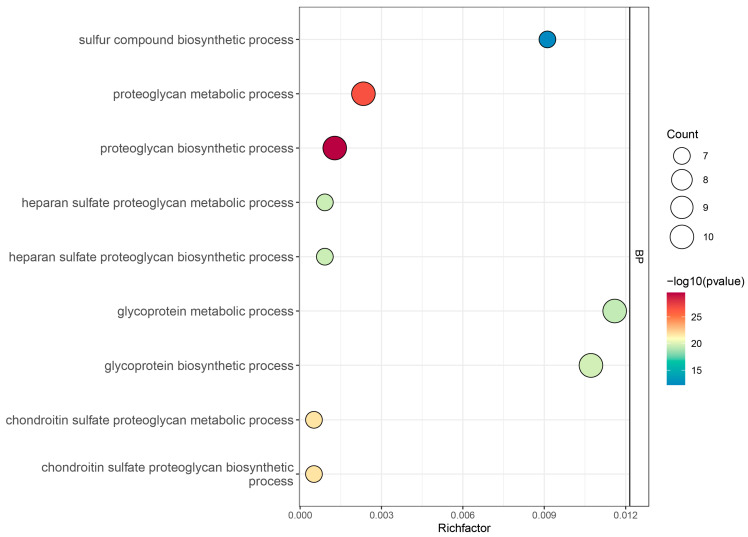
GO enrichment analysis of *ZmGT14* gene family. The *x*-axis represents the enrichment factor (Richfactor), and the *y*-axis lists the enriched GO terms (description). Bubble size corresponds to the number of genes enriched in each term (Count > 4), while the color gradient indicates the significance level (−log10 (*p*-value)), with redder colors representing higher enrichment significance.

To further elucidate the biological functions of *ZmGT14* family members, Gene Ontology (GO) enrichment analysis was performed across three categories (Figure 8, Table S5): molecular function (MF), cellular component (CC), and biological process (BP). At the biological process (BP) level, ZmGT14 genes exhibited a broad functional distribution, with a pronounced association with glycan-related metabolic processes. Notably, these genes showed clear enrichment in proteoglycan and glycoprotein metabolic and biosynthetic processes, highlighting their important roles in the assembly and modification of complex carbohydrates. At the molecular function (MF) level, ZmGT14 genes were mainly associated with UDP-glycosyltransferase activity and glucuronosyltransferase activity, which is consistent with the fundamental biochemical roles of GT14 family members as glycosyltransferases. Regarding the cellular component (CC) category, ZmGT14 proteins were predominantly localized to the Golgi membrane, suggesting that the Golgi apparatus represents a key subcellular site for their functional activity. This localization is in agreement with their involvement in the biosynthesis of non-cellulosic polysaccharides in the plant cell wall.

### 3.10. Expression Analysis of ZmGT14 Gene Family Under Drought Stress

**Figure 9:**
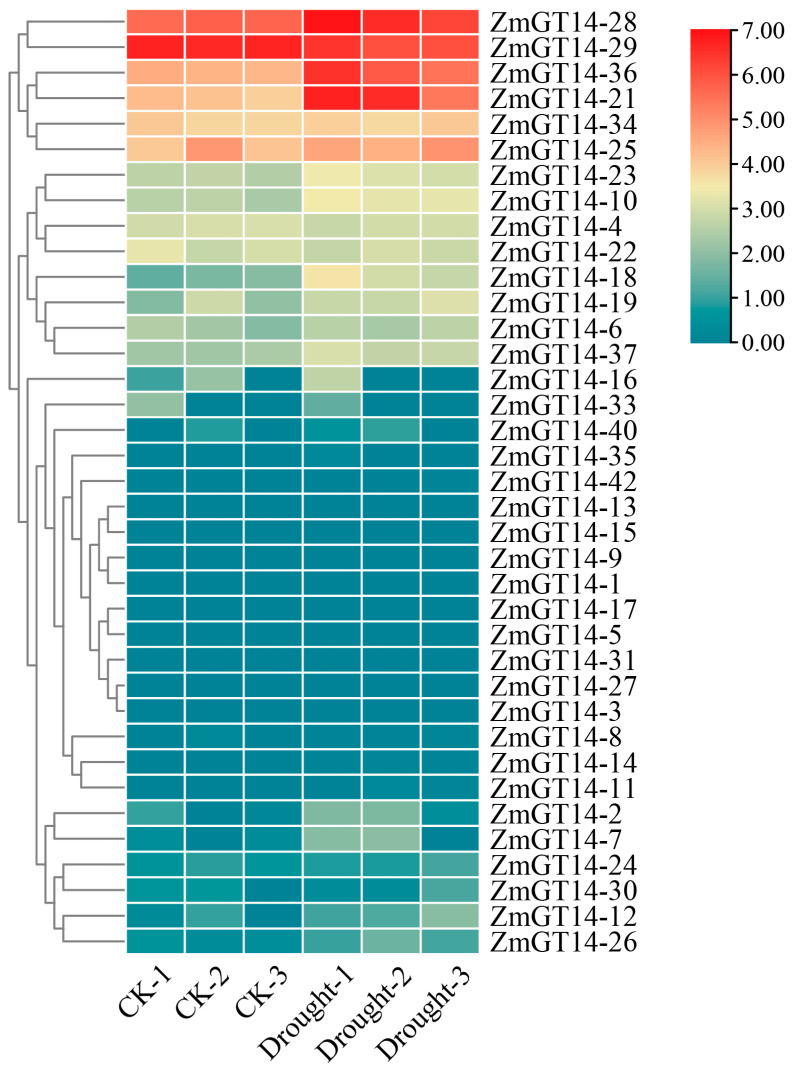
Expression patterns of *ZmGT14* gene family under drought stress. The heatmap shows the transcriptional differences of 42 *ZmGT14* members under control conditions (CK-1/2/3) and drought treatment (Drought-1/2/3). The color gradient represents normalized gene expression levels, with red indicating high expression and blue indicating low expression.

Based on transcriptome data under drought stress, *ZmGT14* family members exhibited distinct differential expression patterns (Figure 9). During drought treatment, *ZmGT14-21*, *28*, *29*, and *36* were strongly upregulated, suggesting a positive regulatory role in maize drought tolerance. In contrast, *ZmGT14-26*, *12*, and most other members maintained low expression levels or were downregulated under the same conditions. This pronounced differential expression under drought stress likely contributes to cell wall remodeling and osmotic protection mechanisms, thereby enhancing maize adaptability to water-deficient environments.

### 3.11. RT-qPCR Validation of ZmGT14 Gene Family Under Drought Stress

**Figure 10:**
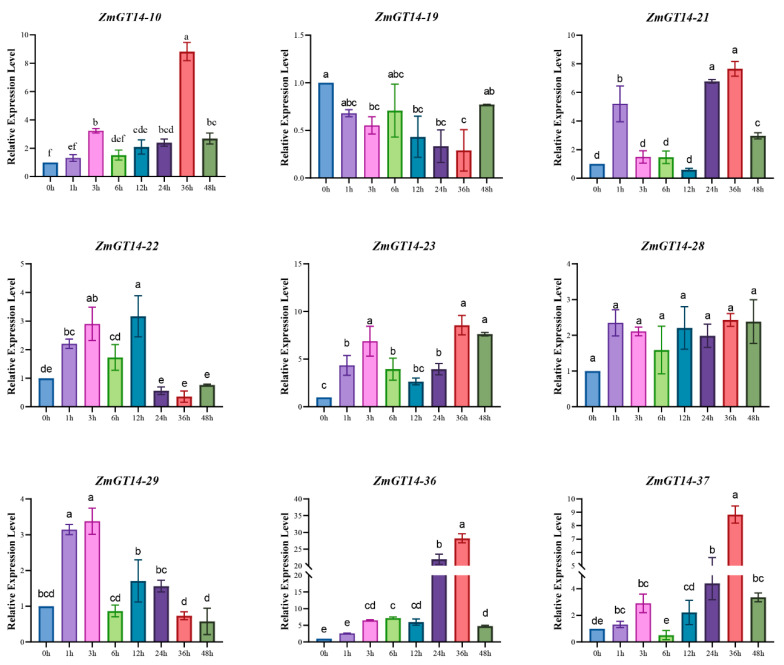
Validation of the expression patterns of nine candidate *ZmGT14* genes under drought stress. The figure shows the relative expression levels of nine key *ZmGT14* members measured by RT-qPCR at different time points of drought treatment (0, 1, 3, 6, 12, 24, 36, and 48 h). The *x*-axis represents the treatment time, and the *y*-axis indicates the relative gene expression levels. Different letters above the bars denote significant differences at *p* < 0.05 (Duncan’s test).

To further validate the transcriptome data and clarify the dynamic responses of *ZmGT14* genes under drought stress, nine representative members were selected for RT-qPCR analysis (Figure 10). The results revealed distinct differential expression patterns of these genes over the 0–48 h drought treatment period. Most of the selected genes exhibited clear drought-induced upregulation. Specifically, *ZmGT14-10*, *21*, *23*, *36*, and *37* displayed a significant increase in expression over time, reaching peak levels at 36 h and slightly declining at 48 h. Among these, *ZmGT14-36* showed the most pronounced response, with peak expression approximately 30-fold higher than at 0 h. *ZmGT14-28* exhibited a sustained induction pattern, rapidly upregulated at 1 h and maintaining high expression across subsequent time points. *ZmGT14-29* and *22* showed early induction, peaking at 3 h and 12 h, respectively, before gradually decreasing, suggesting their roles in early drought signal transduction. In contrast, *ZmGT14-19* was consistently downregulated throughout the treatment, with expression levels remaining below those of the control. Overall, the RT-qPCR results were highly consistent with the transcriptome heatmap trends, indicating that *ZmGT14* family members exhibit pronounced temporal and spatial expression divergence in response to drought stress.

### 3.12. Yeast Library Screening and Validation of ZmGT14-36

**Figure 11:**
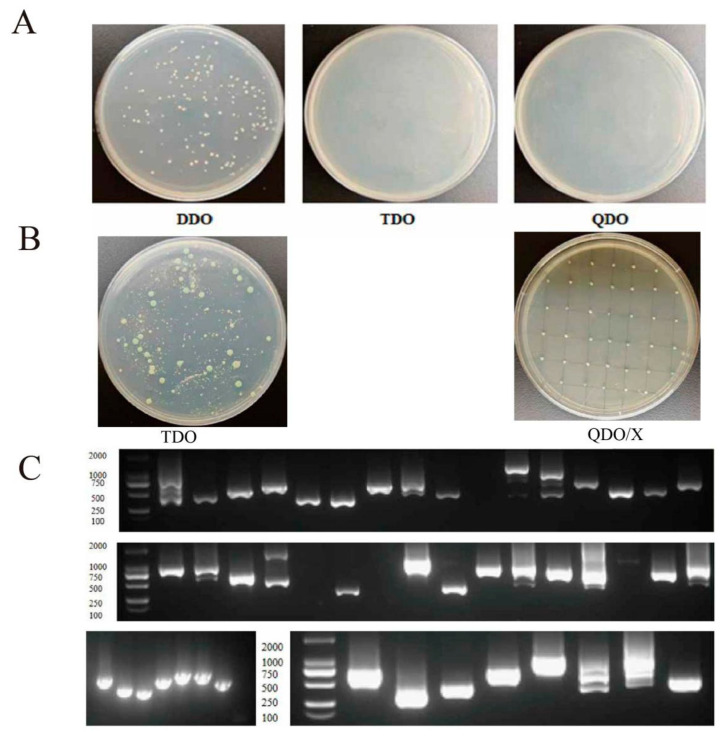
Yeast two-hybrid library screening of ZmGT14-36. (**A**) Auto-activation assay of the bait construct. Yeast cells harboring pGBKT7-ZmGT14-36 grew normally on DDO medium but failed to grow on TDO and QDO media, indicating that ZmGT14-36 lacks auto-activation activity and is suitable for library screening. (**B**) Results of cDNA library co-transformation screening. Left panel shows primary screening on TDO medium; right panel shows secondary screening of positive clones on QDO medium supplemented with X-Gal. (**C**) PCR verification of positive clones from the secondary screening. DNA marker sizes (from top to bottom) are 2000, 1000, 750, 500, 250, and 100 bp.

Consistent with the transcriptome and RT-qPCR results, *ZmGT14-36* exhibited a strong response to drought stress. As plants coordinate responses to abiotic stresses through complex protein regulatory networks, identifying interacting partners of ZmGT14-36 is crucial for elucidating its molecular mechanism. In this study, yeast two-hybrid (Y2H) technology was employed to systematically screen for candidate proteins interacting with ZmGT14-36 in a maize cDNA library. First, autoactivation tests showed that when co-transformed with the empty pGADT7 vector, the bait construct pGBKT7-ZmGT14-36 failed to grow on TDO and QDO selective media, confirming the absence of autoactivation and suitability for subsequent screening (Figure 11A). During library screening, over 500 single clones were obtained on DDO medium. Preliminary screening on TDO medium yielded approximately 500 positive clones (Figure 11B). Subsequently, 50 clones were randomly selected for secondary spot screening on QDO/X medium, and all clones exhibited stable growth (Figure 11B). PCR identification of 48 positive clones revealed that the lengths of the inserted fragments were all greater than 250 bp, mainly ranging from 500 to 1000 bp (Figure 11C), consistent with the expected library insert size distribution.

**Figure 12:**
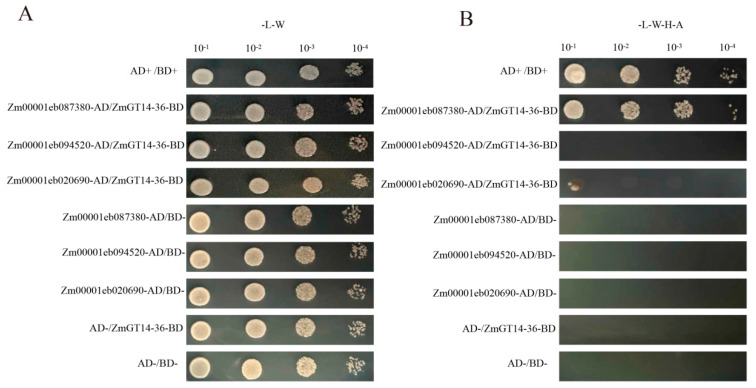
Yeast two-hybrid assay for interaction between ZmGT14-36 and candidate proteins. (**A**) Yeast growth on double dropout (--Leu-–Trp,-–-–W) medium. All co-transformed yeast strains grew normally on-–-–W medium, indicating successful transformation of both the bait vector (ZmGT14-36-BD) and prey vectors (candidate gene–-AD) and the absence of toxicity to yeast cells. (**B**) Interaction analysis on quadruple dropout -–Leu-–Trp-–His/-Ade, -L-W-H-A) medium. The positive control (AD+/BD+) and the combination of Zm00001eb087380-AD with ZmGT14-36-BD showed stable growth on -L-W-H-A medium, demonstrating a clear protein–protein interaction. In contrast, the remaining candidate gene-AD constructs co-transformed with ZmGT14-36-BD, as well as all negative controls, failed to grow on -L-W-H-A medium, indicating the absence of autoactivation or nonspecific interactions. Yeast cultures were serially diluted (10^−1^, 10^−2^, 10^−3^, and 10^−4^) and spotted onto the indicated media.

The identification of these candidate interacting proteins provides important clues for revealing the specific regulatory mechanisms of ZmGT14-36 in maize drought-response networks. Based on agarose gel electrophoresis, PCR products with single and clear bands were selected for sequencing. A total of 36 sequencing results were obtained, and homology comparison was performed using the MaizeGDB database (https://maizegdb.org/, accessed on 10 September 2025). The resulting gene list and annotation analysis are provided in the annotation table (Table S6). According to gene functional annotation and their potential associations with cell wall metabolism and stress response regulation, three candidate genes (Zm00001eb020690, Zm00001eb087380, Zm00001eb330890) were selected for cloning and ligated into the AD vector. Point-to-point validation using the yeast two-hybrid assay revealed that one of these candidates, Zm00001eb087380_T001 (UDP-GLYCOSYLTRANSFERASE 85A2-RELATED, UGT85A2), interacts with ZmGT14-36 (Figure 12).

## 4. Discussion

In this study, we identified 42 members of the *ZmGT14* gene family. Based on PFAM domain analysis, these members share conserved domains consistent with those found in Arabidopsis and rice, including *GT14* and *GT14-like* domains, and are predicted to possess glycosyltransferase activity involved in AGP biosynthesis [23,45]. Phylogenetic and synteny analyses revealed high collinearity and close evolutionary relationships between maize and rice, suggesting that *ZmGT14* members may have retained biological functions similar to their rice homologs in cell wall modification. Previous studies in rice have shown that mutations in *BC10* and *OsGT14;1* alter cellulose and arabinose content in the cell wall, thereby affecting cell wall mechanical strength [20,25]. Our protein–protein interaction (PPI) predictions further indicated potential cooperative interactions among *GT14* family members. Such synergistic patterns have been reported in other GT families, such as the *GT8* family (e.g., the *GAUT1*–*GAUT7* complex) [46]. This cooperative behavior may enhance enzyme stability and catalytic efficiency, enabling maize to efficiently complete complex cell wall biosynthesis tasks, especially during cell wall remodeling under environmental stress conditions.

In this study, transcriptome sequencing combined with RT-qPCR validation revealed that *ZmGT14-36* exhibits a dramatic upregulation under drought stress, with expression increasing up to 30-fold. Such pronounced expression dynamics suggest that this gene may act at an upstream or key regulatory node in the plant’s stress defense response. Consistent with its promoter enrichment in ABRE (abscisic acid-responsive element) and DRE (drought-responsive element), the strong induction of *ZmGT14-36* indicates its critical role in maize’s response to drought.

Promoter analysis further showed that *ZmGT14* promoters contain multiple stress-responsive elements, including ABRE, DRE, LTR, and MYB/MYC, as well as hormone-responsive elements such as AuxRE, SARE, and numerous ABREs, suggesting responsiveness to auxin, salicylic acid, and abscisic acid signals. Notably, yeast two-hybrid library screening identified the auxin response factor *IAA31* as an interacting partner. Previous studies in *Betula platyphylla* reported that *GT14;6* can interact with WRKY or ARF transcription factors [47], and the identification of IAA31 suggests that ZmGT14-36 may occupy a hub at the intersection of hormonal signaling. Thus, the *GT14* family not only participates in structural polysaccharide synthesis but may also modulate auxin signaling to reallocate growth resources under stress, balancing development and stress adaptation [48].

This study confirmed a direct physical interaction between ZmGT14-36 and UGT85A2. *UGT85A2* is a highly conserved and functionally versatile gene in plants, primarily belonging to the *UGT85* family. It catalyzes the addition of glucose molecules to specific substrates, facilitating the biosynthesis of secondary metabolites with flavor or color and modulating hormone activity to cope with environmental stresses [49,50,51]. In Arabidopsis, *UGT85A2* is known to enhance drought tolerance by reducing reactive oxygen species (ROS) accumulation and regulating root development [52]. According to the “calcium capacitor” model proposed by Lopez-Hernandez [19], GT14 enzymes add glucuronic acid (GlcA) residues to AGP side chains, and these negatively charged residues serve as a physical basis for calcium ion retention in the cell wall. Under drought stress, the upregulation of *ZmGT14-36* in maize may increase GlcA modification of AGPs, thereby strengthening the cell wall’s capacity to buffer and store Ca^2+^. This not only maintains cell wall structural integrity under water deficit but also provides the material basis for calcium-mediated signaling cascades triggered by environmental stress.

In this study, combined transcriptomic and RT-qPCR data preliminarily revealed the potential involvement of *ZmGT14-36* in the response to drought stress. Although yeast two-hybrid (Y2H) assays identified a candidate interaction between ZmGT14-36 and UGT85A2, this relationship requires further validation through in vivo and in vitro biochemical experiments, such as BiFC and Pull-down assays, to account for the inherent limitations and potential false positives of the Y2H system. Furthermore, due to the current lack of genetic evidence from loss-of-function mutants or overexpression lines, the precise molecular mechanism by which *ZmGT14-36* regulates stress resistance remains to be fully elucidated. Future research will focus on genetic transformation experiments to construct and refine the *ZmGT14-36* mediated regulatory network under drought stress.

## 5. Conclusions

In summary, this study systematically identified the *ZmGT14* gene family in maize, revealing its high conservation in evolutionary relationships, gene structure, and conserved motifs. Comprehensive analyses of *cis*-acting elements, miRNA regulatory networks, and GO functional enrichment highlighted the potential roles of *ZmGT14* members in regulating cell wall metabolism and responding to stress signals. Transcriptome sequencing and RT-qPCR results demonstrated that *ZmGT14* family members are significantly induced under drought stress and exhibit differential expression patterns, with *ZmGT14-36* showing a strong positive regulatory response. Yeast two-hybrid experiments further confirmed the physical interaction between ZmGT14-36 and UGT85A2. These findings establish *ZmGT14-36* as a positive regulator in maize drought response, providing valuable candidate gene resources and theoretical support for breeding drought-tolerant maize varieties.

## Data Availability

The original contributions presented in this study are included in the article/Supplementary Materials. Further inquiries can be directed to the corresponding authors.

## Supplementary Materials

The following supporting information can be downloaded at: https://www.mdpi.com/article/10.3390/plants15030512/s1, Table S1. Primers used for *ZmGT14* gene amplification. Table S2. Physicochemical properties of the *ZmGT14* gene family. Table S3. Whole-genome duplication (WGD) gene pairs of the *ZmGT14* gene family. Table S4. Zm*GT14* exon and intron number. Table S5. Functional Enrichment Analysis for ZmGT14. Table S6. Functional annotation of genes identified from yeast library screening of ZmGT14-36.
