# Supplementary material for: Genome-Wide Identification and Characterization of the Maize ZmGT14 Gene Family Reveals ZmGT14-36 as a Drought-Responsive Gene Interacting with UGT85A2
Source: Plants (Basel). 2026 Feb 6;15(3):512. doi: 10.3390/plants15030512 (PMC12900031; doi:10.3390/plants15030512)
Supplement: Supplementary file 1 [file plants-15-00512-s001.zip › plants-4100862-supplementary.pdf]

Table S1. Primers used for *ZmGT14* gene amplification

Primer name	Sequence (5'–3')
ZmGT14-10-F	GGATATGAAGGCCGTCCCAC
ZmGT14-10-R	AGCCTACCAGGAAGCCAATC
ZmGT14-19-F	CGTGAACTCCCCCTCCTACG
ZmGT14-19-R	CTGTCGCTGACGATGCTACC
ZmGT14-23-F	GTTTCATTGATTGCTTCTTCGATCC
ZmGT14-23-R	AGCTATTCCACCAGGGTCAA

ZmGT14-21-F	GGTCGTGAGGGATATGCGAC
ZmGT14-21-R	CACTCAGGAGAAAACTTGCGG
ZmGT14-22-F	TCTGAGAGTTGTGTGCCACT
ZmGT14-22-R	GTAAAGGGTGTCCGCGAGAA
ZmGT14-28-F	AATGCGACGACTGAGGAAGG
ZmGT14-28-R	GCCTTCTTGTGTGAGCAAGC
ZmGT14-29-F	GCTACATCGAGGTGCTGGAG
ZmGT14-29-R	GTCGGGAAGTAGTGCTCCTC
ZmGT14-36-F	GATTGACCAGGAACTGTTGGC
ZmGT14-36-R	TCAACCGTGAAGGGTTTCCC
ZmGT14-37-F	GAAAGGGTGCCCAGTGGTTT
ZmGT14-37-R	CATGGAAGTTCTCATCAACTGCT
ZmGT14-BD-EcoRI-F	CATGGAGGCCGAATTCATGCTAGAGGCGAAGCCG
ZmGT14-BD-BamHI-R	GCAGGTCGACGGATCCCAGGCAATGCTTGTCATCAAAAC
Zm00001eb020690-AD-ECOR1-F	GGAGGCCAGTGAATTCATGGGCGGTGGCAAC
Zm00001eb020690-AD-BAMHI-R	CGAGCTCGATGGATCCCTTCTTCAGATGGGGGAAGCAC
Zm00001eb087380-AD-ECORI-F	GGAGGCCAGTGAATTCATGGGTTCGCTGCCGC
Zm00001eb087380-AD-BAMHI-R	CGAGCTCGATGGATCCGGCTAGGAGCACCTCTTCGAT
Zm00001eb330890-AD-ECORI-F	GGAGGCCAGTGAATTCATGGACGGCGGATACTACG
Zm00001eb330890-AD-BAMHI-R	CGAGCTCGATGGATCCGTAGTAGGGCTGGATCTTCCAGC
ZmActin1-F	TACGAGATGCCTGATGGTCAGGTCA
ZmActin1-R	TGGAGTTGTACGTGGCCTCATGGAC

Table S2. Physicochemical properties of the *ZmGT14* gene family

Gene_ID	Name	Protein Length	CDS Length (nt)	Molecular Weight (Da)	Isoelectric Point (pI)	Hydrophobicity (GRAVY)	Localizations
Zm00001eb003670	ZmGT14-1	405	1215	44644.66	6.53	-0.01	Endoplasmic Reticulum
Zm00001eb012530	ZmGT14-2	239	717	27126.97	9.17	-0.32	Extracellular
Zm00001eb032020	ZmGT14-3	341	1023	37842.22	9.77	-0.02	Golgi
Zm00001eb033150	ZmGT14-4	417	1251	46099.92	6.49	-0.12	Golgi
Zm00001eb047520	ZmGT14-5	399	1197	43847.58	11.6	-0.64	Extracellular
Zm00001eb053580	ZmGT14-6	455	1365	49489	8.9	-0.07	Golgi
Zm00001eb056180	ZmGT14-7	229	687	26569.95	6.22	-0.35	Extracellular
Zm00001eb056190	ZmGT14-8	403	1209	45757.02	6.73	-0.19	Golgi
Zm00001eb056200	ZmGT14-9	160	480	18589.9	5.26	-0.37	Nucleus
Zm00001eb071400	ZmGT14-10	387	1161	45223.51	8.84	-0.34	Golgi
Zm00001eb100910	ZmGT14-11	247	741	26883.42	8.84	-0.03	Golgi
Zm00001eb120610	ZmGT14-12	463	1389	50704.09	9.72	-0.18	Golgi
Zm00001eb125170	ZmGT14-13	403	1209	45423.53	9.63	-0.33	Golgi
Zm00001eb125190	ZmGT14-14	403	1209	45609.04	9.05	-0.23	Golgi
Zm00001eb125810	ZmGT14-15	347	1041	38400.36	8.96	-0.06	Golgi
Zm00001eb134140	ZmGT14-16	394	1182	43264.03	9.75	-0.15	Golgi
Zm00001eb146060	ZmGT14-17	430	1290	48195.35	8.99	-0.22	Golgi
Zm00001eb155920	ZmGT14-18	391	1173	44559.08	9.79	-0.25	Endoplasmic Reticulum
Zm00001eb204130	ZmGT14-19	449	1347	49382.75	9.98	-0.25	Golgi
Zm00001eb206910	ZmGT14-20	388	1164	44791.25	8.82	-0.18	Golgi

Gene_ID	Name	Protein Length	CDS Length (nt)	Molecular Weight (Da)	Isoelectric Point (pI)	Hydrophobicity (GRAVY)	Localizations
Zm00001eb218590	ZmGT14-21	360	1080	41129.95	7.22	-0.14	Golgi
Zm00001eb226850	ZmGT14-22	372	1116	43135.01	8.28	-0.26	Golgi
Zm00001eb233490	ZmGT14-23	377	1131	43422.68	8.71	-0.18	Golgi
Zm00001eb238210	ZmGT14-24	464	1392	50639.12	9.76	-0.26	Golgi
Zm00001eb260460	ZmGT14-25	414	1242	47053.95	8.04	-0.22	Golgi
Zm00001eb272940	ZmGT14-26	374	1122	43239.17	8.78	-0.29	Golgi
Zm00001eb274600	ZmGT14-27	287	861	32979.31	6.12	-0.34	Cytoplasm
Zm00001eb276760	ZmGT14-28	441	1323	49163.51	6.96	-0.13	Endoplasmic Reticulum
Zm00001eb300280	ZmGT14-29	351	1053	38538.76	8.37	-0.1	Golgi
Zm00001eb305740	ZmGT14-30	144	432	17072.12	6.2	-0.3	Cytoplasm
Zm00001eb309330	ZmGT14-31	455	1365	51009.02	10.14	-0.5	Endoplasmic Reticulum
Zm00001eb334920	ZmGT14-32	402	1206	45267.21	9.05	-0.16	Golgi
Zm00001eb345880	ZmGT14-33	332	996	38286.27	9.3	-0.42	Endoplasmic Reticulum
Zm00001eb354090	ZmGT14-34	421	1263	46688.92	9.02	-0.06	Golgi
Zm00001eb370070	ZmGT14-35	376	1128	40140.65	6	-0.16	Endoplasmic Reticulum
Zm00001eb385590	ZmGT14-36	439	1317	49086.54	7.3	-0.15	Golgi
Zm00001eb389650	ZmGT14-37	378	1134	43472.83	7.97	-0.18	Golgi
Zm00001eb397620	ZmGT14-3	407	1221	45834.09	9.47	-0.29	Golgi

Gene_ID	Name	Protein Length	CDS Length (nt)	Molecular Weight (Da)	Isoelectric Point (pI)	Hydrophobicity (GRAVY)	Localizations
	8						
Zm00001eb414090	ZmGT14-39	370	1110	43652.65	8.84	-0.36	Golgi
Zm00001eb416550	ZmGT14-40	269	807	29404.34	5.37	-0.1	Golgi
Zm00001eb430510	ZmGT14-41	284	852	33156.42	6.56	-0.39	Cytoplasm
Zm00001eb434580	ZmGT14-42	437	1311	49032.24	9.21	-0.21	Golgi

Table S3. Whole-genome duplication (WGD) gene pairs of the *ZmGT14* gene family

Chromosome	Gene ID	Chromosome	Gene ID	Duplication Type
chr1	ZmGT14-2	chr9	ZmGT14-38	WGD
chr1	ZmGT14-3	chr5	ZmGT14-21	WGD
chr10	ZmGT14-39	chr2	ZmGT14-10	WGD
chr10	ZmGT14-41	chr2	ZmGT14-10	WGD
chr4	ZmGT14-19	chr5	ZmGT14-24	WGD
chr5	ZmGT14-21	chr7	ZmGT14-29	WGD
chr5	ZmGT14-22	chr6	ZmGT14-26	WGD
chr4	ZmGT14-20	chr5	ZmGT14-23	WGD
chr5	ZmGT14-23	chr6	ZmGT14-27	WGD
chr5	ZmGT14-23	chr9	ZmGT14-37	WGD
chr6	ZmGT14-28	chr9	ZmGT14-36	WGD
chr4	ZmGT14-20	chr9	ZmGT14-37	WGD
chr6	ZmGT14-27	chr9	ZmGT14-37	WGD

Table S4. Zm*GT14* exon and intron number

**gene**	**Exon_number**	**Intron_number**
ZmGT14-1	5	4
ZmGT14-2	3	2
ZmGT14-3	1	0
ZmGT14-4	9	8
ZmGT14-5	5	4
ZmGT14-6	5	4
ZmGT14-7	2	1
ZmGT14-8	2	1
ZmGT14-9	4	3
ZmGT14-10	11	10
ZmGT14-11	2	1
ZmGT14-12	3	2
ZmGT14-13	2	1
ZmGT14-14	2	1
ZmGT14-15	5	4
ZmGT14-16	4	3
ZmGT14-17	2	1
ZmGT14-18	11	10
ZmGT14-19	2	1
ZmGT14-20	11	10
ZmGT14-21	2	1
ZmGT14-22	11	10
ZmGT14-23	11	10
ZmGT14-24	2	1
ZmGT14-25	2	1
ZmGT14-26	11	10
ZmGT14-27	14	13
ZmGT14-28	4	3
ZmGT14-29	2	1
ZmGT14-30	3	2
ZmGT14-31	10	9
ZmGT14-32	5	4
ZmGT14-33	12	11
ZmGT14-34	4	3
ZmGT14-35	3	2
ZmGT14-36	4	3
ZmGT14-37	11	10
ZmGT14-38	5	4
ZmGT14-39	11	10
ZmGT14-40	5	4
ZmGT14-41	13	12
ZmGT14-42	2	1

Table S5. Functional Enrichment Analysis for ZmGT14

ONTOLOGY	ID	Description	Count	pvalue	p.adjust
BP	GO:0030166	proteoglycan biosynthetic process	10	2.77E-30	1.33E-28
BP	GO:0006029	proteoglycan metabolic process	10	2.29E-27	5.49E-26
BP	GO:0050650	chondroitin sulfate proteoglycan biosynthetic process	7	1.78E-22	2.13E-21
BP	GO:0050654	chondroitin sulfate proteoglycan metabolic process	7	1.78E-22	2.13E-21
BP	GO:0009101	glycoprotein biosynthetic process	10	1.72E-20	1.65E-19
BP	GO:0015012	heparan sulfate proteoglycan biosynthetic process	7	2.49E-20	1.71E-19
BP	GO:0030201	heparan sulfate proteoglycan metabolic process	7	2.49E-20	1.71E-19
BP	GO:0009100	glycoprotein metabolic process	10	3.81E-20	2.29E-19
BP	GO:0044272	sulfur compound biosynthetic process	7	5.64E-13	3.01E-12
BP	GO:0048679	regulation of axon regeneration	3	4.18E-09	1.43E-08
BP	GO:0048681	negative regulation of axon regeneration	3	4.18E-09	1.43E-08
BP	GO:0070570	regulation of neuron projection regeneration	3	4.18E-09	1.43E-08
BP	GO:0070571	negative regulation of neuron projection regeneration	3	4.18E-09	1.43E-08
BP	GO:1903035	negative regulation of response to wounding	3	4.18E-09	1.43E-08
BP	GO:0031102	neuron projection regeneration	3	7.67E-09	2.16E-08
BP	GO:0031103	axon regeneration	3	7.67E-09	2.16E-08
BP	GO:0048678	response to axon injury	3	7.67E-09	2.16E-08
BP	GO:1903034	regulation of response to wounding	3	4.63E-08	1.23E-07
BP	GO:0010977	negative regulation of neuron projection development	3	8.00E-08	1.92E-07
BP	GO:0031345	negative regulation of cell projection organization	3	8.00E-08	1.92E-07

BP	GO:0045665	negative regulation of neuron differentiation	3	9.04E-08	1.97E-07
BP	GO:0050768	negative regulation of neurogenesis	3	9.04E-08	1.97E-07
BP	GO:0070555	response to interleukin-1	3	1.72E-07	3.59E-07
BP	GO:0031099	regeneration	3	2.27E-07	4.54E-07
BP	GO:0010721	negative regulation of cell development	3	2.69E-07	5.17E-07
BP	GO:0061564	axon development	3	6.78E-07	1.25E-06
BP	GO:0051961	negative regulation of nervous system development	3	2.23E-06	3.97E-06
BP	GO:0045596	negative regulation of cell differentiation	3	3.16E-06	5.41E-06
BP	GO:0010975	regulation of neuron projection development	3	3.39E-06	5.62E-06
BP	GO:0045664	regulation of neuron differentiation	3	4.75E-06	7.60E-06
BP	GO:0031344	regulation of cell projection organization	3	6.24E-06	9.35E-06
BP	GO:0120035	regulation of plasma membrane bounded cell projection organization	3	6.24E-06	9.35E-06
BP	GO:0050767	regulation of neurogenesis	3	6.98E-06	1.02E-05
BP	GO:0032102	negative regulation of response to external stimulus	3	1.14E-05	1.60E-05
BP	GO:0034097	response to cytokine	3	1.31E-05	1.78E-05
BP	GO:0031175	neuron projection development	3	1.33E-05	1.78E-05
BP	GO:0051960	regulation of nervous system development	3	1.55E-05	2.02E-05
BP	GO:0048666	neuron development	3	2.14E-05	2.71E-05
BP	GO:0030182	neuron differentiation	3	3.28E-05	4.04E-05
BP	GO:0048699	generation of neurons	3	4.16E-05	4.99E-05
BP	GO:0022008	neurogenesis	3	5.55E-05	6.50E-05
BP	GO:0120036	plasma membrane bounded cell projection organization	3	7.22E-05	8.25E-05

BP	GO:0030030	cell projection organization	3	7.87E-05	8.79E-05
BP	GO:0060284	regulation of cell development	3	1.17E-04	1.28E-04
BP	GO:0007399	nervous system development	3	1.47E-04	1.57E-04
BP	GO:0080135	regulation of cellular response to stress	3	1.56E-04	1.63E-04
BP	GO:0045595	regulation of cell differentiation	3	5.25E-04	5.36E-04
BP	GO:0051129	negative regulation of cellular component organization	3	5.42E-04	5.42E-04
CC	GO:0000139	Golgi membrane	3	5.76E-06	5.76E-06
MF	GO:0015020	glucuronosyltransferase activity	3	7.54E-09	1.51E-08
MF	GO:0008194	UDP-glycosyltransferase activity	3	4.35E-06	4.35E-06

Table S6. Functional annotation of genes identified from yeast library screening of ZmGT14-36

Gene	Pfam	GO	Functional annotation
Zm00001eb094520_T002	PF00627 PF00632 PF06012 PF06025	GO:0004842; GO:0005515	E3 ubiquitin-protein ligase HUWE1
Zm00001eb020690_T001	PF14377 PF04419 PF12907	—	ZF-MET2 domain-containing protein
Zm00001eb087380_T001	PF00201	GO:0008194	UDP-glycosyltransferase 85A2-related
Zm00001eb157400_T001	PF01775	GO:0003735; GO:0005840; GO:0006412	Large subunit ribosomal protein L18Ae
Zm00001eb198420_T001	PF01781	GO:0003735; GO:0005840; GO:0006412	Large subunit ribosomal protein L38e
Zm00001eb241920_T001	PF03009	GO:0006629; GO:0008081	Glycerophosphocholine phosphodiesterase
Zm00001eb058590_T001	PF08041	GO:0009512	Cytochrome b6-f complex subunit 7
Zm00001eb332360_T001	PF02309	GO:0005515	Auxin-responsive protein IAA31
Zm00001eb140320_T001	PF00141	GO:0004601; GO:0006979; GO:0020037	Peroxidase
Zm00001eb380850_T001	PF05627	—	RPM1-interacting protein 4 family protein
Zm00001eb096370_T001	PF00112	GO:0006508; GO:0008234	Senescence-specific cysteine protease SAG12
Zm00001eb194020_T001	PF04674	—	Protein EXORDIUM
Zm00001eb355990_T001	PF00280	GO:0004867; GO:0009611	Serine protease inhibitor (potato inhibitor I-type)
Zm00001eb313850_T001	PF00847	GO:0003677; GO:0003700; GO:0006355	Ethylene-responsive transcription factor RAP2-12
Zm00001eb288390_T001	PF13516	GO:0005515	F-box protein SKP2A
Zm00001eb330890_T001	—	—	Ricin B-like lectins
Zm00001eb244200_T001	PF08122	GO:0005739; GO:0022900	NADH dehydrogenase [ubiquinone] 1 beta subcomplex subunit 3
Zm00001eb001950_T001	PF14159	—	Protein curvature thylakoid 1A, chloroplastic
Zm00001eb370530_T001	PF07818	GO:0006355	SAP30-binding protein
